# Intraventricular Hemorrhage Severity as a Predictor of Outcome in Intracerebral Hemorrhage

**DOI:** 10.3389/fneur.2019.00217

**Published:** 2019-03-12

**Authors:** Gabriela Trifan, Baback Arshi, Fernando D. Testai

**Affiliations:** Department of Neurology and Rehabilitation, University of Illinois at Chicago College of Medicine, Chicago, IL, United States

**Keywords:** IVH, Graeb score, outcome, spontaneous ICH, supratentorial hemorrhage

## Abstract

**Background/Objective:** Intraventricular hemorrhage (IVH) extension after spontaneous supratentorial intracerebral hemorrhage (sICH) is an independent predictor of worse outcome. However, there is a paucity of data looking at the degree of IVH severity and its impact on outcome. This study addresses the contribution of IVH severity to outcome at time of hospital discharge after sICH.

**Methods:** Two hundred and ten patients were included in the study. Baseline demographic and radiologic characteristics were abstracted. First available CT scans were reviewed for hematoma volume and location, IVH extension and presence of hydrocephalus (HCP). IVH severity was calculated using Graeb scale. Multivariate logistic regression models were developed to investigate the association of IVH severity with poor outcomes at hospital discharge, defined as modified Rankin scale score (mRS) >3.

**Results:** Fifty-three percent of patients had IVH extension while 18% had surgical procedures done. Poor outcome (mRS >3) was seen for 56% of patients. Median IVH extension severity on the Graeb scale was two. Presence of IVH was associated with poor outcome in univariate and multivariate analysis (*p* < 0.005). Compared to patients with no IVH, IVH severity influenced outcome only when Graeb scores were ≥5 (OR = 1.3, 95% CI 0.49–3.23, *p* = 0.63, and OR = 2.9, 95% CI, 1.1–7.6, *p* = 0.03 for Graeb <5 and ≥5, respectively.

**Conclusions:** Higher IVH severity (defined as Graeb score ≥5) is associated with worse outcome at time of hospital discharge, while lower IVH severity (Graeb scores 1–4) has similar outcomes to patients without IVH. IVH severity should be used in favor of IVH presence for prognostication purposes.

## Introduction

Spontaneous intracerebral hemorrhage (sICH) has an overall incidence of 24.6 per 100,000 patient-years and the incidence increases with age (1). sICH accounts for 10–15% of all strokes and is characterized by a 30–50% 3-month mortality rate (2–4). Intraventricular hemorrhage (IVH) extension can be seen in up to 54% of sICH and is an independent predictor of worse outcome and neurological deterioration (4–13). IVH extension is included as a variable in the calculation of the ICH score. The ICH score is a prognostic tool commonly used in clinical practice. This includes age ≥80 years, Glasgow coma scale (GCS), infratentorial location, hematoma volume, and presence of IVH. The ICH scores ranges from 0 to 6, with 30-day mortality rates increasing from 0% for a score of 0 to 100% for score of 5 or 6 (11). In the ICH score, IVH extension is indicated as present or absent regardless of severity (8, 12). While it is already established that IVH extension is independently associated with high mortality and poor functional outcome, the impact of the degree of IVH severity is not completely understood. The objective of this study is to determine the contribution of IVH severity to outcome after sICH.

## Materials and Methods

**Figure 1:**
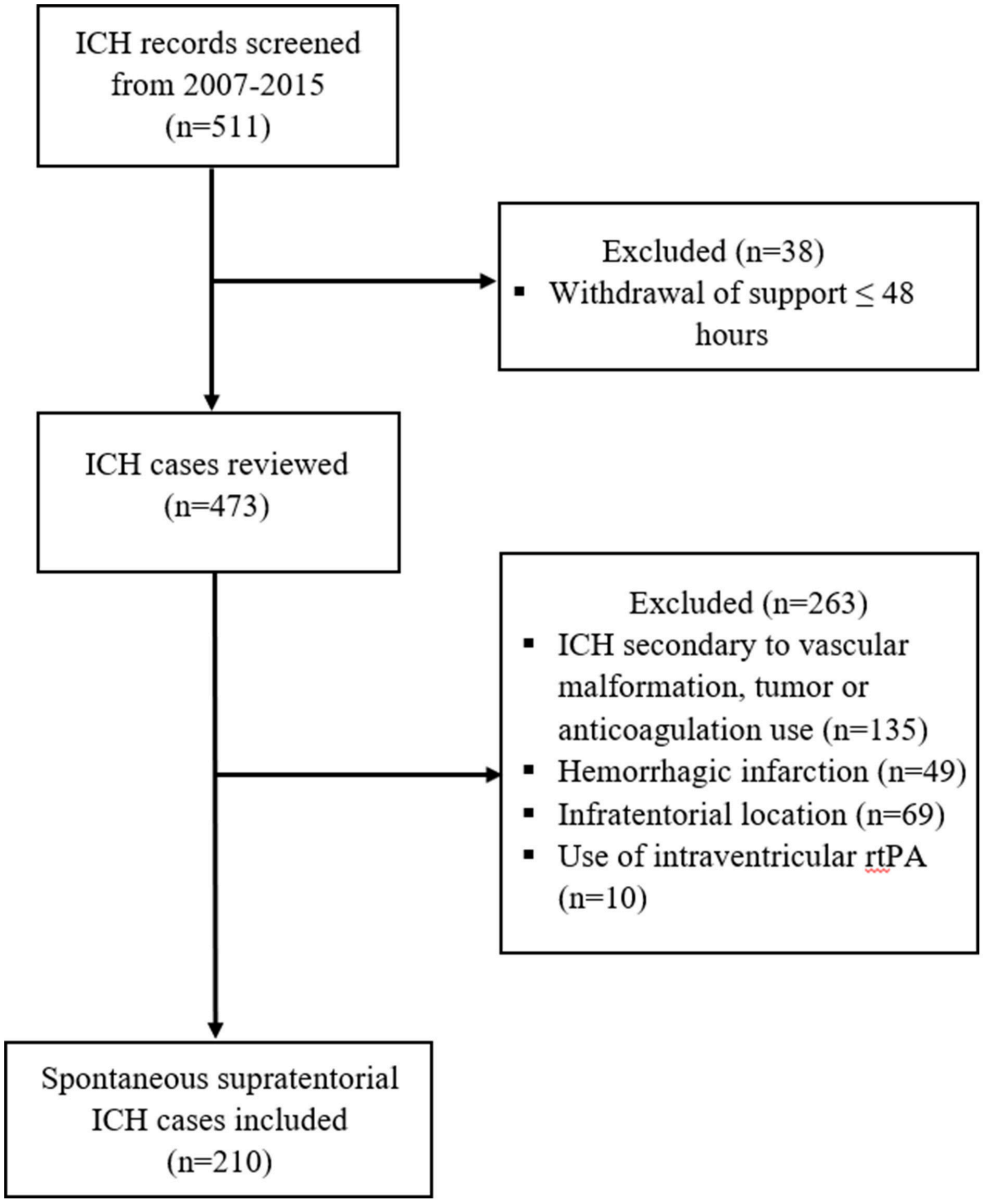
Flow chart of inclusion and exclusion criteria.

This study is a single-center retrospective analysis of patients with sICH admitted to a tertiary care center from 2007 to 2015. This study was approved by the local Institutional Review Board and follows the guidelines set forth by the Strengthening the Reporting of Observational Studies in Epidemiology (STROBE) statement. Electronic medical records were screened for discharge diagnosis of ICH using ICD-9 and ICD-10 codes. We included patients aged older than 18 with spontaneous sICH, which was defined as parenchymal hemorrhage that was not related to tumor, vascular malformations, or trauma. Exclusion criteria were hemorrhagic infarction, anticoagulation associated ICH, and intraventricular treatment with recombinant activated tissue plasminogen activator (Figure 1). Early restriction of care in patients with anticipated poor outcome might lead to self-fulfilling prophecy and introduce bias in predictive models (14). Thus, we also excluded patients with withdrawal of support within the 48 h of admission.

### Clinical and Radiologic Information

Sociodemographic information, medical history, baseline GCS, surgical procedures, and outcome at hospital discharge were abstracted from the electronic medical records. Hematoma volume was determined using the ABC/2 formula (15). ICH score was calculated as previously described (11). The severity of IVH extension was calculated using the Graeb scale (16). The Graeb scale is a semiquantitative tool for IVH extension scoring, ranging from 0 to 12 points, with higher scores denoting increased IVH volumes; a maximum of four is given for each lateral ventricle if expanded and filled with blood and up to 2 for the third and fourth ventricle if filled with blood and expanded (16). Hydrocephalus (HCP) was assessed using the score proposed by Diringer et al. (17). In this score, the frontal horn, atrium, and temporal horn of each lateral ventricle as well as the third and fourth ventricles are individually assigned a 0, 1, 2, or points for no, mild, moderate, or marked HCP, respectively. This score ranges from 0 to 24 with higher scores indicating worse HCP.

The radiologic variables were determined by research personnel blinded to the outcomes using the first available non-contrast computed tomography (CT) of the head. Functional outcome was assessed using modified Rankin scale (mRS) at time of hospital discharge. mRS was dichotomized into good outcome (0–3 points) and poor outcome (4–6 points) (18).

### Statistical Analysis

Descriptive variables are presented as percentages, mean ± standard deviation (SD), or median and interquartile range (IQR). Fisher Exact test and Mann Whitney test were used to compare categorical and continuous variables, respectively. Logistic regression analysis was used to identify predictors of poor outcome at hospital discharge. Variables included in the statistical analysis were chosen based on their known influence on outcome. ICH volume and Graeb score were initially analyzed as continuous variables. Logistic regression analyses were performed to determine the association of different variables of interest with outcome. Two different adjustment models were utilized. In model 1, the results were adjusted by the components of the ICH score (age, GCS, and ICH volume). In model 2, the results were adjusted by variable characteristics with p < 0.05 in univariate analysis. Patients with IVH extension were then subcategorized based on the degree of IVH severity using the Graeb scale. Outcomes at hospital discharge for each Graeb score were compared to patients without IVH extension using logistic regression. The Graeb score cut point for poor outcome was identified and used to group patients based on IVH severity. Multivariate logistic regression analyses for groups using the Graeb score cut point were performed adjusting by the two proposed models.

Presence of multicollinearity among outcome and independent variables was assessed using weighted regression. Multicollinearity was defined as variance inflation factor ≥5 or tolerance of <0.20 (19). Statistical analyses were done using SPSS version 24 (SPSS Inc., Chicago, Il) with *p* < 0.05 indicating statistical significance.

## Results

A total of 511 cases were screened and 210 met the inclusion criteria (Figure 1). Baseline demographic, radiologic, and clinical characteristics are shown in Table 1. The large majority of the patients (98%) had first ever sICH and 46% had documented history of hypertension. Hematoma affected the deep brain structures in 64% of cases. History of antiplatelet medication use was seen in 9% of cases. IVH extension was present in 53% of patients with sICH, and of these, the median Graeb score was 2. Hydrocephalus was observed in 48% of patients and surgical hematoma evacuation was performed in 20 cases (10%). All the patients that required VP shunt placement (8%) were in the ICH with IVH extension group. Poor outcome (mRS >3) at hospital discharge was seen in 56% of all patients.

**Table 1:** Characteristics.

**Variables**	**Participants (*N* = 210)**
Age mean ±*SD*	56 ± 13
Male gender, *n* (%)	120 (57%)
GCS score at baseline (median, IQR)	14 (9–15)
ICH volume, mL (median, IQR)	20 (6–38)
IVH extension, *n* (%)	112 (53)
Graeb score (median, IQR)	2 (0–7)
Hydrocephalus score (median, IQR)	0 (0–8)
**Hematoma location**, ***n*** **(%)**	
Deep	134 (64)
Lobar	76 (36)
Antiplatelet use at baseline *n* (%)	18 (9)
**Past medical history** ***n*** **(%)**	
Diabetes	36 (17)
Hypertension	134 (64)
Previous ICH	4 (2)
Ischemic stroke	22 (10)
**Surgical procedure type**, ***n*** **(%)**	
VP shunt placement	17 (8)
Clot evacuation	20 (10)
mRS score at discharge median (IQR)	4 (2–5)

*sICH, spontaneous intracerebral hemorrhage; IVH, intraventricular hemorrhage; VP shunt, Ventriculoperitoneal shunt; mRS, Modified Rankin Scale score*.

Multicollinearity was not observed between the independent variables studied and outcome. In univariate analysis, history of hypertension (HTN), history of diabetes (DM), GCS score, hematoma volume, Graeb score, hydrocephalus score on admission, and clot evacuation, were all associated with poor outcome (*p* < 0.05) (Table 2). In model 1, age, hematoma volume, GCS score, and Graeb score were independently associated with poor outcome; in model 2, only GCS score, hematoma volume and Graeb score remained significantly associated with poor outcome (Table 3). When analyzing the effect of each degree of IVH extension severity on outcome at time of hospital discharge, only Graeb scores ≥5 were significantly associated with poor outcome (OR = 12, 95% CI, 1.4–102.3, *p* = 0.025) (Table 4). In multivariate analysis adjusted for model 1, subjects with Graeb score <5 had similar outcomes to individuals with no IVH extension (OR = 1.5, 95% CI, 0.5–4.0, *p* = 0.4), while patients with Graeb score ≥5, had a 4-fold increase risk for mRS>3, compared with no IVH extension patients (OR = 4.0, 95% CI, 1.6–10.1, *p* = 0.003) (Table 5). Similar findings were seen after adjusting for variables in model 2: patients with IVH extension severity as measured on Graeb scale ≥5, were three times more likely to have poor outcome at time of hospital discharge compared with sICH patients without IVH extension (OR = 2.9, 95% CI, 1.11–7.59, *p* = 0.03) (Table 5).

**Table 2:** Univariate analysis for predictors of poor outcome.

	**Univariate**	
**Variable**	**OR (95% CI)**	***P*-value**
Age	1.01 (1.00–1.03)	0.1
Male gender	1 (0.58–1.74)	1
History of HTN	0.6 (0.31–0.97)	0.04
History of DM	2.7 (1.19–6.02)	0.02
Antithrombotic use	1.6 (0.58–4.40)	0.37
GCS score	0.70 (0.59–0.76)	<0.0001
sICH volume	1.06 (1.04–1.08)	<0.0001
^*^IVH extension	1.38 (1.25–1.53)	<0.0001
Hydrocephalus score	1.14 (1.07–1.20)	<0.0001
Hematoma location	0.8 (0.44–1.36)	0.38
VP shunt placement	3.4 (0.99–13.03)	0.051
Clot evacuation	0.3 (0.95–0.92)	0.035

*HTN, Hypertension; DM, Diabetes Mellitus; GCS, Glasgow coma scale; sICH, spontaneous intracerebral hemorrhage*.

* *As measured by Graeb scale*.

**Table 3:** Multivariate analysis for predictors of poor outcome (mRS >3).

**Variable**	**Model 1**		**Model 2**	
	**OR (95% CI)**	***P* value**	**OR (95% CI)**	***P* value**
Age	1.03 (1.00–1.06)	0.03	1.03 (1.01–1.06)	0.02
GCS score	0.76 (0.65–0.88)	<0.0001	0.76 (0.66–0.88)	<0.0001
sICH vol	1.05 (1.03–1.07)	<0.0001	1.05 (1.02–1.07)	<0.0001
^*^IVH extension	1.21 (1.07–1.36)	0.002	1.16 (1.02–1.31)	0.02
Hydrocephalus score	1.05 (0.98–1.14)	0.12	1.05 (0.97–1.31)	0.20
Clot evacuation	0.98 (0.12–4.92)	0.98	0.80 (0.16–3.91)	0.79
History of HTN	0.61 (0.28–1.32)	0.21	0.77 (0.35–1.69)	0.52
History of DM	1.46 (0.48–4.43)	0.51	1.38 (0.46–4.02)	0.57

*HTN, Hypertension; DM, Diabetes Mellitus; GCS, Glasgow coma scale; sICH, spontaneous intracerebral hemorrhage*.

* *As measured by Graeb scale. In model 1 outcomes were adjusted by the ICH score variables (age, GCS, hematoma volume, and IVH extension). In model 2 outcomes were adjusted by variables that had p ≤ 0.05 in the univariate analysis*.

**Table 4:** Odds for poor outcome (modified Rankin Scale>3) based on Graeb scores.

**Graeb score**	***N***	**OR (95% CI)**	***P*-value**
0	98	*Reference*	–
1	5	0.5 (0.1–4.6)	0.534
2	10	1.2 (0.5–7.2)	0.310
3	7	4.9 (0.9–26.8)	0.065
4	8	1.2 (0.5–8.3)	0.359
5	7	12 (1.4–102.3)	0.025
6	21	6.3 (2.1–18.7)	0.001
7	12	21.7 (2.7–175.1)	0.004
8	8	13.8 (1.6–116.8)	0.016
9	7	11.8 (1.4–102.3)	0.025
10	12	21.6 (2.7–175.1)	0.004
11	10	17.7 (2.2–145.9)	0.008
12^∧^	5	–	–

∧ *Unable to calculate due to no patients with good outcome*.

**Table 5:** Odds for poor outcome (modified Rankin Scale>3) based on grouped Graeb scores.

**Graeb score**	***n***	**Unadjusted**		**Model 1**		**Model 2**	
		**OR (95% CI)**	***P*-value**	**OR (95% CI)**	***P*-value**	**OR (95% CI)**	***P*-value**
1–4	30	1.9 (0.86–4.51)	0.1	1.5 (0.57–4.01)	0.410	1.3 (0.49–3.23)	0.63
5–12	82	12.7 (5.94–27.20)	<0.0001	4.0 (1.59–10.15)	0.003	2.9 (1.11–7.59)	0.03

**Figure 2:**
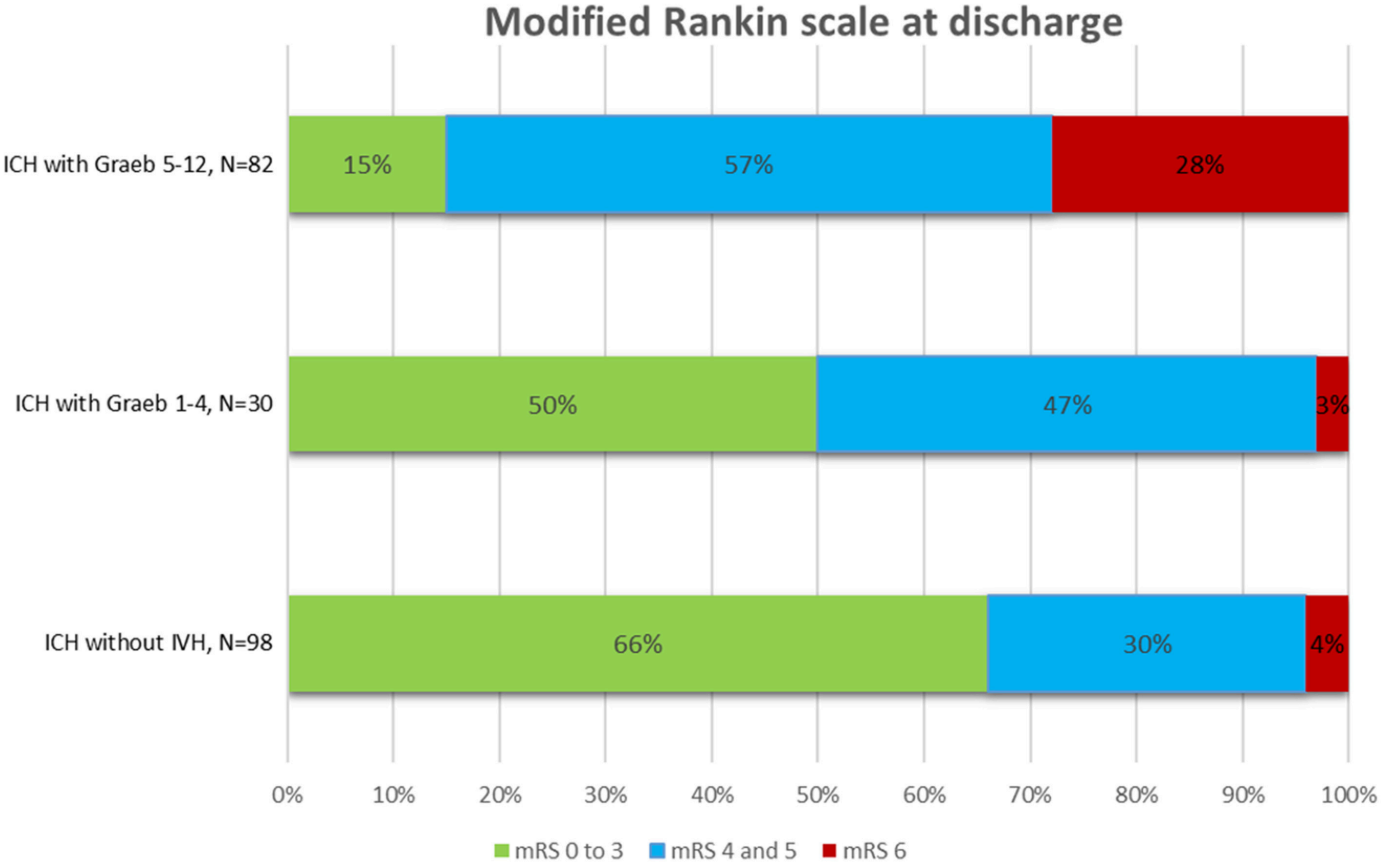
Outcome at hospital discharge after intracerebral hemorrhage (ICH) with and without intraventricular hemorrhage (IVH).

The overall mortality rate was 13%. sICH patients with IVH extension had higher mortality rates (21%) compared with patients without IVH extension (4%), *p* < 0.0001. Among patients with IVH extension, the mortality rates increased from 3% in patients with Graeb scores <5, to 28% for those with scores ≥5, *p* < 0.0001 (Figure 2).

## Discussion

The results of our study show that Graeb score ≥5 after sICH is an independent predictor of poor outcome at time of hospital discharge. In contrast, patients with sICH and Graeb scores <5 had similar outcomes to the sICH patients without IVH extension.

To date, the presence of IVH extension after supratentorial sICH is a known independent predictor of poor outcome (10, 12, 20–23). However, the contribution of IVH severity to outcome has not been thoroughly investigated. Tuhrim et al. was the first to demonstrate that IVH volume is associated with higher 30-day mortality rates (10). Young et al. identified a threshold volume of 20 mL as being predictive of poor outcome (20). However, IVH extension volumes as low as 6 mL have been shown to increase the likelihood of poor functional outcome after sICH (24). Performing volumetric analysis requires imaging reprocessing software that is not typically available in clinical practice. This drawback has been circumvented by the use of semiquantitative IVH extension severity scales which estimate IVH extension volume by using visual grading systems.

Two independent groups showed that IVH extension severity, measured as a continuous variable by the modified Graeb scale, is associated with poor outcome in sICH (6, 25). These studies demonstrated that IVH extension severity scales can refine the accuracy of our prediction models but did not identify a severity threshold that could be used for prognostication purposes.

Three studies investigating the association of pre-specified cut-off values with outcome showed mixed results for IVH extension severity. In one of these studies, Graeb score ≥5 was associated with increased functional impairment at hospital discharge (9). In another study, increasing IVH severity estimated using the Graeb scale with patients divided into quartiles correlated with decreased survival and functional independence (6). In contrast, another study of 153 patients with sICH showed that increasing IVH extension severity measured using Graeb score divided into tertiles was not significantly associated with 30-day mortality or disability at 6-months (23). These studies are summarized in Table 6. A direct comparison of our study with those presented in Table 6 is prevented by the exclusion of sICH cases with infratentorial location in this series, variations in the outcome measures, differences in follow-up, and other methodological details. However, all studies, including ours, confirm that IVH extension volume is a key contributor to patient outcomes just like hematoma volume. In particular our study provides a unique perspective by showing outcomes data for each degree of IVH extension severity and identifies an IVH extension severity cutoff of Graeb ≥5 as a predictor of poor neurologic outcome. Our study also highlights that the use of IVH extension as a dichotomized variable decreases the accuracy of the prognostication tools commonly used in clinical practice.

**Table 6:** Overview of selected studies in spontaneous intracerebral hemorrhage with intraventricular hemorrhage.

**Study**	**Type of study *N* (% IVH)**	**Outcome scale**	**Follow-up (months)**	**Results**	**Notes**
**ICH WITH/WITHOUT IVH (NO SEVERITY GRADING)**					
Tuhrim et al. (10)	Prospective 129 (36%)	Mortality rates	1	42.5 vs. 8.5%	No cutoff identified
Steiner et al. (21)	Prospective 374 (45%)	Poor functional outcome (mRS ≥4)	3	56.7 vs. 24.5%	No cutoff identified
Bhattathiri et al. (22)	Prospective 902 (42%)	Favorable functional outcome (GOS ≥4)	6	31vs. 15% (*p* < 0.00001), when IVH absent vs. present	No cutoff identified
Hallevi et al. (7)	Retrospective 406 (45%)	Poor functional outcome (mRS ≥4)	3	OR = 3.4, 95% CI = 2.21–5.09 (*p* < 0.0001)	No cutoff identified
Bhatia et al. (12)	Prospective 214 (48%)	Mortality predictor	Hospital discharge	OR = 2.66, 95% CI = 1.31–5.41 (*p* = 0.007)	No cutoff identified
Mustanoja et al. (13)	Prospective 967 (41%)	Mortality rates	3	54 vs. 18% (*p* < 0.001)	No cutoff identified
**ICH WITH/WITHOUT IVH (GRADED BASED ON SEVERITY)**					
Morgan et al. (25)	Databank 1,250 (32%)	Poor functional outcome (mRS ≥3)	3	Increasing modified Graeb scores correlate with poor outcome. OR = 1.12, 95% CI = 1.05–1.16 (*p* < 0.0001)	Modified Graeb Score used as a continuous variable No cutoff identified
**ICH WITH/WITHOUT IVH (GRADED BASED ON DEFINED SEVERITY CUTOFFS)**					
Godoy et al. (23)	Prospective 153 (48%)	Rate of favorable functional outcome (GOS ≥4)	6	36.4 vs. 14.7% vs. 16.7% for Graeb scores of 1–4, 5–8, and 9–12, respectively (*p* = 0.20)	Graeb score 1–4 vs. 5–8 vs. 9–12 Included patients with infratentorial and supratentorial location
Nishikawa et al. (9)	Retrospective 100 (35%)	Functional outcome (KPS 50–100)	hospital discharge	OR = 3.96, 95% CI = 0.90–17.38; (*p* = 0.068)	Graeb score ≤ 5 vs. >5
Hansen et al. (6)	Prospective 198 (43%)	Survival and poor functional outcome (mRS ≥4)	1 and 3	Increasing modified Graeb scores correlate with 30 days survival, OR = 1.16; 95% CI = 1.06–1.27 (*p* = 0.002) and poor outcome OR = 1.11, 95% CI = 1.02–1.20 (*p* = 0.011)	Survival was analyzed by modified Graeb scores divided into quartiles: the third and fourth quartiles had worse survival vs. first and second quartiles

*GOS, Glasgow Outcome Score; KPS, Karnofsky Performance Status Scale; IVH, intraventricular hemorrhage; mRS, modified Rankin Scale*.

Our study has some limitations, including the relatively small sample size of patients with IVH extension. However, the sample size of our study is larger compared with previously reported studies that looked at IVH extension severity and outcome. Second, functional outcome using mRS was determined at time of hospital discharge. Although 3- and 6-month outcomes are preferable to report functional outcome, these data were not readily available for our cohort. Third, our study included only patients with supratentorial sICH. Thus, these results cannot be extrapolated to infratentorial ICH or ICH resulting from vascular malformations, tumor, or trauma. Fourth, in this study we used the Graeb score and did not look into the predictive value of other semiquantitative tools such as the modified Graeb score, IVH score, or LeRoux score. Increased IVH extension severity, measured by any of these scores correlates with poor outcome (24, 25). However, additional studies are necessary to determine the severity cut-off points for these scoring systems.

In conclusion, we showed that increased IVH extension severity, defined by a Graeb score ≥5, is an independent predictor of poor outcome at hospital discharge after sICH. When using risk stratification tools for clinical severity grading after sICH, IVH extension should be given different weights based on the amount of IVH extension present, rather than being used as a dichotomized variable.

## Data Availability

The datasets generated for this study are available on request to the corresponding author.

## Author Contributions

FT and GT contributed conception and design of the study. GT organized the database and wrote the first draft of the manuscript. GT and FT performed the statistical analysis. GT, FT, and BA wrote sections of the manuscript. All authors contributed to manuscript revision, read, and approved the submitted version.

### Conflict of Interest Statement

The authors declare that the research was conducted in the absence of any commercial or financial relationships that could be construed as a potential conflict of interest.
